# Diaper rashes can indicate systemic conditions other than diaper dermatitis

**DOI:** 10.1186/s12895-020-00104-z

**Published:** 2020-09-21

**Authors:** Sirirus Lebsing, Jitjira Chaiyarit, Leelawadee Techasatian

**Affiliations:** 1 Pediatric Department, Dermatology Division, Faculty of Medicine, Khon Kaen University, Khon Kaen, 40002 Thailand; 2 Clinical epidemiology unit, Faculty of Medicine, Khon Kaen University, Khon Kaen, Thailand

**Keywords:** Diaper rash, Diaper dermatitis, Differential diagnosis

## Abstract

**Background:**

Although the majority of rashes in the diaper area are caused by irritation from urine and feces, irritant diaper dermatitis; IDD, there are some less common but potentially serious cutaneous eruptions associated with systemic diseases that should not be discounted.

**Methods:**

This prospective descriptive study aimed to explore variation in cutaneous disease in the diaper area. It was conducted as a prospective descriptive study between October 2016 and November 2019 in the pediatric department of a tertiary-level hospital.

**Results:**

Three hundred consecutive patients with rashes in the diaper area were enrolled. The most common diagnosed was IDD (125 cases; 41.7%), followed by rashes exacerbated by the diaper (101 cases; 33.67%) and non-diaper-related rashes (74 cases; 24.67%).

**Conclusions:**

Our finding suggests that when diagnosing rashes that occur in the diaper area, general pediatricians should consider, in addition to IDD, the possibility of less-common conditions. The simultaneous presence of cutaneous lesions at other sites was linked to diagnoses of systemic diseases other than IDD, (*P* < 0.001).

## Background

Rashes around the diaper area are common in the pediatric population, especially among those who are diaper dependent [1–3]. The majority of rashes in the diaper area turn out to be irritant diaper dermatitis (IDD), meaning that they are caused by irritation from urine and feces, which is aggravated by wearing diapers. This type of skin inflammation is mostly found in diaper-dependent children under 24 months of age [3]. Although IDD is the most common diagnosis in cases of inflammation in the diaper area, there are some less common but sometimes serious cutaneous eruptions associated with systemic diseases that should not be discounted. Coughlin el al [4]. classified skin conditions that present in the diaper area in to three groups: 1) skin conditions caused by the presence of the diaper, 2) rashes exacerbated by the diaper (but not directly caused by it), and 3) eruptions present regardless of the presence of the diaper. Previous reviews have indicated a variety of differential diagnoses of cutaneous eruptions in the diaper area [4–8]. However, there are no statistical data available regarding the frequencies of various types of cutaneous eruptions in the genitoanal area. We thus attempted to ascertain such data using a prospective descriptive study design. Our aim was to promote recognition of some less common diseases in the diaper area in order to prevent misdiagnosis and to identify cutaneous clues indicating systemic diseases that are likely to be misdiagnosed as IDD.

## Methods

### Study design and participants

This was a prospective descriptive study exploring variation in cutaneous diseases in the diaper area conducted between October 2016 and November 2019 in the pediatric department of a single tertiary-level hospital (Khon Kaen University, Thailand). The study was approved by the institutional review board of the Khon Kaen University Human Ethical Committee and conducted in accordance with the Declaration of Helsinki (#HE591334). The study was funded by a grant from the Khon Kaen University Faculty of Medicine in Thailand (Grant Number IN62334).

All patients under 18 years of age were eligible. Consecutive patients with rashes in the diaper area who presented at the pediatric department (both in outpatient and inpatient settings) were asked to participate in the study. A total of 300 children were enrolled according to the formula for determination of sample size for estimating proportions. The estimated proportion (p) of IDD in the pediatric population was 0.25 based on the results of a previous study [1]. We aimed for a confidence coefficient of 95% and used an absolute precision (d) of 0.05. The standard normal z-value for a significance level a = 0.05, which was 1.96.

Before participants were enrolled in the study, written informed consent regarding the use of all images for a medical publication was obtained from their parents or guardians.

### Measurement used

Rashes in the diaper area were diagnosed clinically by a pediatric dermatologist with some supportive investigation, such as tissue staining, blood testing, and tissue pathology, as necessary. The demographic background information collected included age, sex, general condition, initial presenting symptoms, diaper use, other underlying diseases, other sites of cutaneous eruption, and photographs of uncommon rashes in the diaper area.

### Statistics

At the end of the study, the collected data were analyzed using STATA version 10 (StataCorp LP). Descriptive statistics – means, standard deviations (SDs), medians, and frequencies – were used to analyze the demographic data.

A Fisher’s exact test was used for the analysis of categorical data and comparison between groups. Statistical significance was determined based on the exact approach under the Bonferroni correction. *P* values < 0.05 were considered significant.

## Results

A total of 300 patients who presented with rashes in the diaper area were recruited in-to the study. Participants’ ages ranged from 2 weeks to − 168 months, with a median age of 6 months (IQR 2–29). One hundred thirty-four (44.67%) of the patients were male, and 166 (55.33%) were female. Two hundred fifteen (71.67%) were outpatient cases, and the remaining 85 (28.33%) were recruited from an inpatient setting.

One hundred seventy patients with diaper rashes (56.7%) initially presented with dermatological problems, while the rest presented with problems involving other organ systems, and the diaper rashes were recognized afterward. These included gastrointestinal (66 cases; 22%), infectious (24 cases; 8%), respiratory (17 cases; 5.7%), and neurological problems (12 cases; 4%).

The most common diagnosis was IDD (125 cases; 41.7%), followed by intertrigo (39 cases; 13%), candidiasis (25 cases; 8.3%), eczema (21 cases; 7%), and seborrheic dermatitis (18 cases; 6%). Other less common cutaneous lesions in the diaper area were psoriasis, impetigo, infantile hemangioma, hand-foot-mouth disease, acrodermatitis enteropathica, staphylococcal scalded skin syndrome (SSSS), Langerhans cell histiocytosis (LCH), scabies, Henoch Sherleiin Purpura (HSP), Stevens Johnson syndrome (SJS), Lichen Sclerosus et Atrophicus (LS&A), warts, Kawasaki disease, bullous pemphigoid, and epidermolysis bullosa.

The authors classified rashes around the diaper area into three types; 1) skin conditions caused by the presence of the diaper, 2) rashes exacerbated by the diaper (but not directly caused by it), and 3) eruptions present regardless of the presence of the diaper. The first was the most common type in this study, of which IDD was the only diagnosis (125 cases, 41.67%), and the second and third were found in 101(33.67%) and 74 cases (24.67%), respectively. See Table 1 for list of skin rashes found along with the numbers of cases.

**Table 1:** Rashes in the diaper area in the study population classified into three groups

Rashes in the diaper area and disease diagnosis	Number cases (%)
**1) Skin condition caused by the presence of the diaper**	
Irritant diaper dermatitis (IDD)	125 (41.67)
**2) Rashes exacerbated by the diaper (but not directly caused by it)**	
Intertrigo	39 (13)
Candida albicans diaper dermatitis	25 (8.33)
Seborrheic dermatitis	18 (6)
Psoriasis	10 (3.33)
Impetigo (Streptococcal/ Staphylococcal infection)	9 (3)
**3) Eruptions present regardless of the presence of the diaper**	
Eczema	21 (7)
Hemangioma	12 (4)
Hand foot mouth disease	7 (2.33)
Acrodermatitis enteropathica	6 (2)
Staphylococcal scalded skin syndrome (SSSS)	6 (2)
Langerhans cell histiocytosis (LCH)	5 (1.67)
Scabies	5 (1.67)
Henoch Sherleiin Purpura (HSP)	4 (1.33)
Steven Johnson syndrome	2 (0.67)
Lichen Sclerosus et Atrophicus (LS&A)	2 (0.67)
Wart	1 (0.33)
Kawasaki disease	1 (0.33)
Bullous pemphigoid	1 (0.33)
Epidermolysis bullosa	1 (0.33)

Factors that differed significantly by type were age at onset of rash, the use of a diaper, and the presence of concurrent skin lesions at sites other than in the diaper area (Table 2).

**Table 2:** Factors associated with type of diaper rashes

Factors	Skin condition caused by the presence of the diaper (***n*** = 125)	Rashes exacerbated by the diaper (but not directly caused by it) (***n*** = 101)	Eruptions present regardless of the presence of the diaper (***n*** = 74)	***p***-value
Sex				< 0.001
Male	37 (29.60)	62 (61.39)	35 (47.30)	
Female	88 (70.40)^a^	39 (38.61)^b^	39 (52.70)^ab^	
Age (months)				< 0.001
age < 24	103 (82.40)^a^	71 (70.30)^ab^	38 (51.35)^b^	
age > = 24	22 (17.60)	30 (29.70)	36 (48.65)	
Age (months)				< 0.001
age < 12	90 (72.00)^a^	69 (68.32)^ab^	34 (45.95)^b^	
age > = 12 - < 24	13 (10.40)	2 (1.98)	4 (5.41)	
age > = 24	22 (17.60)^a^	30 (29.70)^ab^	36 (48.65)^b^	
Diaper use	(*n* = 124)	(*n* = 98)	(n = 74)	< 0.001
No	33 (26.61)^ab^	15 (15.31)^a^	35 (47.30)^b^	
Yes	91 (73.39)	83 (84.69)	39 (52.70)	
Other site of skin lesion				< 0.001
No	124 (99.20)^a^	90 (89.11)^b^	17 (22.97)^c^	
Yes	1 (0.80)	11 (10.89)	57 (77.03)	

Different superscripts (a, b, c, ab) in the same row indicate a significant difference between groups (chi-squared test, multiple comparison test by Bonferroni, *p* < 0.05)

## Discussion

**Fig. 1:**
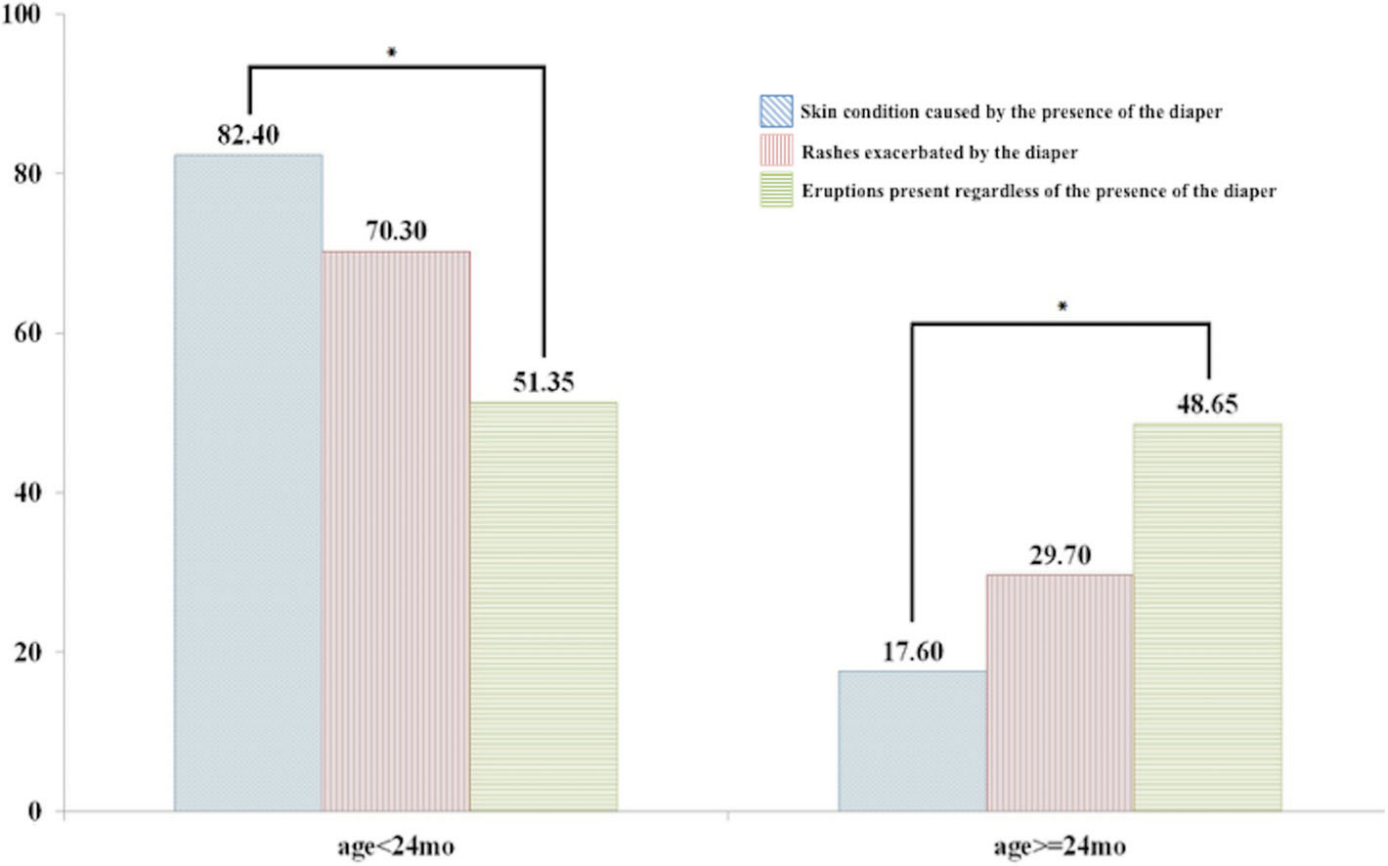
Skin conditions caused by the presence of a diaper were significantly more common than non-diaper related eruptions in participants aged under 24 months, P < 0.001

**Fig. 2:**
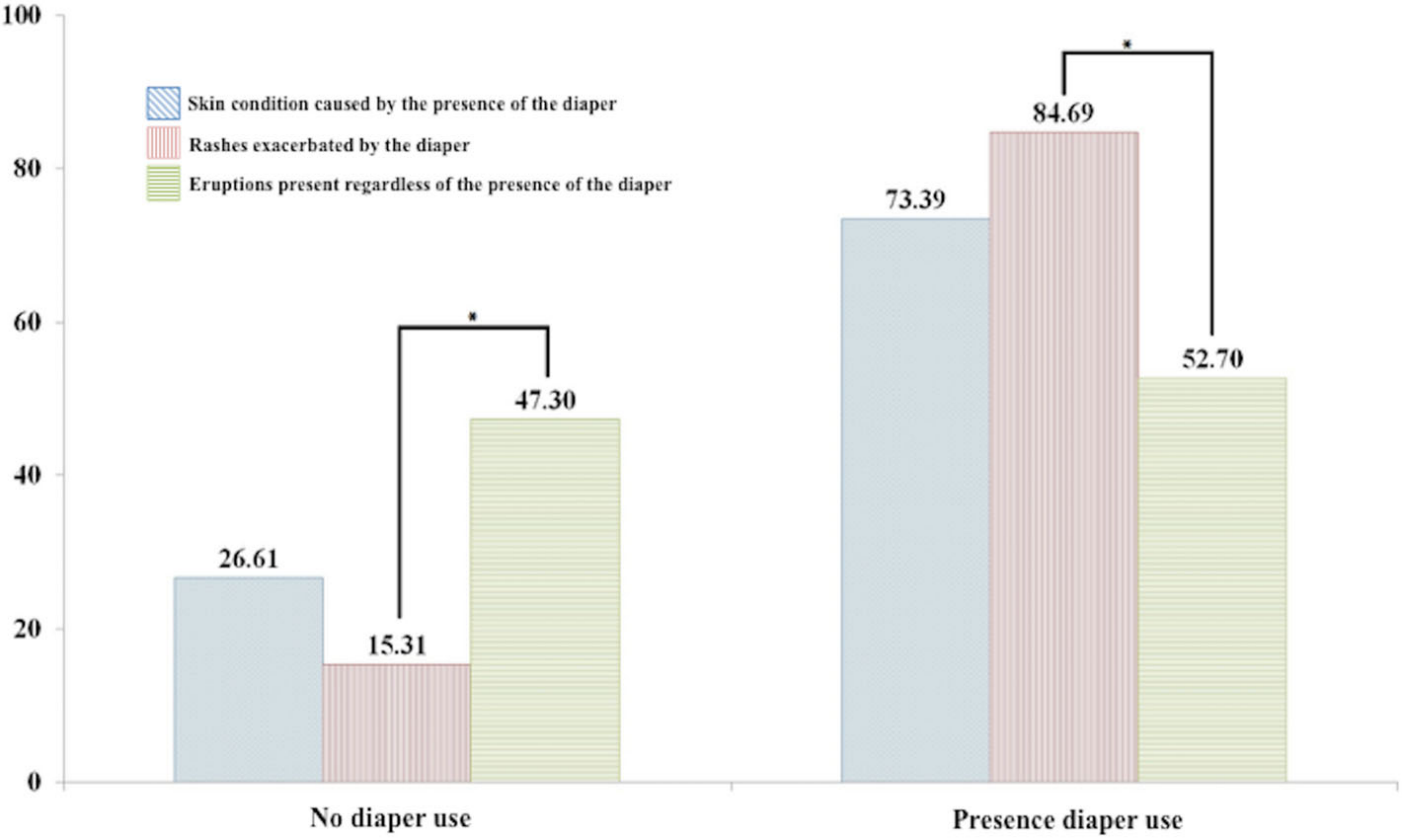
Diaper rashes by group. Significant differences were found between groups in terms of rashes exacerbated by the diaper and eruptions present regardless of the presence of the diaper, *P* < 0.001

We found various types of diaper area rashes in the study population. The most common was IDD, which was found in 41.67% of participants (see Table 1). This type of cutaneous lesion has been defined by Coughlin el al [4] as a skin condition caused by the presence of a diaper. This condition was significantly more common than non-diaper related eruptions in participants aged under 24 months. However, the opposite was true in participants aged over 24 months (*P* < 0.001: Fig. 1), which is likely explained by the fact that children under 24 months of age are more likely to be diaper dependent. We also found significant differences between these two groups in terms of rashes exacerbated by the diaper and eruptions present regardless of the presence of the diaper (Fig. 2). This suggests that non-diaper -related rashes can be present even in diaper-dependent patients. Thus, the differential diagnosis of diaper-independent diseases should be considered in patients not using a diaper [9–12].

**Fig. 3:**
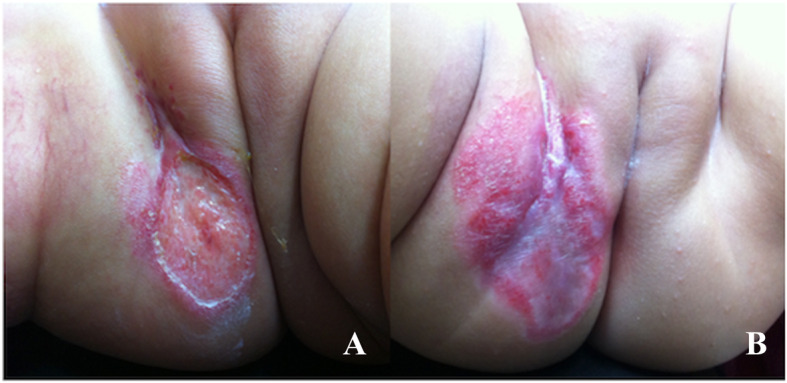
Ulcerated infantile hemangioma in the diaper area (**a**). The cutaneous lesion had improved and ulceration had apparently healed after 1 month of oral propranolol treatment (**b**)

Although the most common rash in the diaper area was IDD, rashes exacerbated by the diaper (but not directly caused by it), and those present independent of diaper use were found in 33.67 and 24.67% cases, respectively. This finding suggests that when rashes occur in the diaper area, general pediatricians should consider less -common rashes as differential diagnoses. In one case in our study, the patient was initially diagnosed with IDD before a visit to a dermatology clinic, at which the condition was diagnosed as an ulcerated hemangioma (Fig. 3a). The first manifestation of the condition was a shallow abrasion, which appeared to be an erosive lesion caused by the moisture and heat that results from diaper use. The patient was thus treated for IDD for 1 month without improvement. However, there was noticeable improvement 1 month after a pediatric dermatologist prescribed oral propranolol as treatment for infantile hemangioma [13] in combination with local wound care (Fig. 3b).

**Fig. 4:**
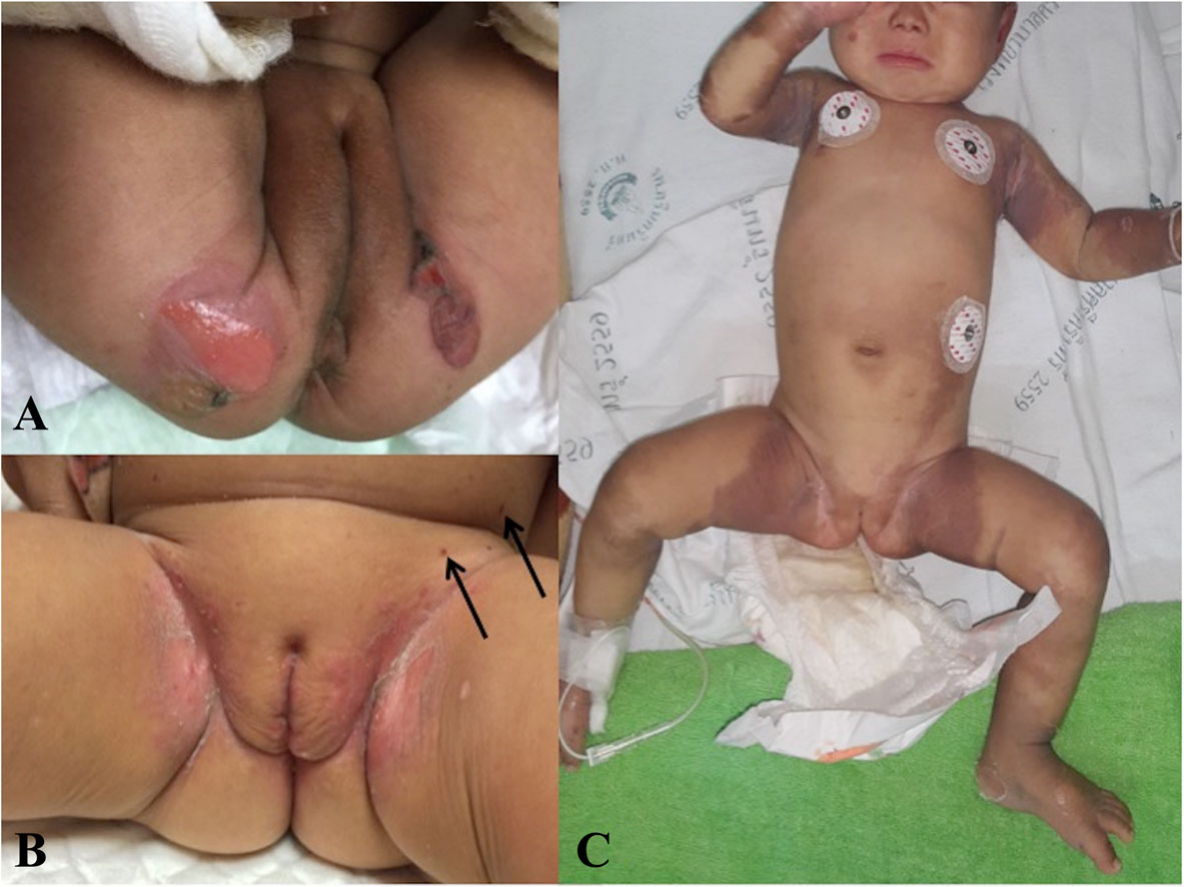
Erosive skin lesions in the diaper area of a patient diagnosed with epidermolysis bullosa (**a**); cutaneous lesion in Langerhans cells histiocytosis (LCH). The arrows indicate petechiae/ hemorrhagic lesions in addition to diaper rashes, the presence of which was a diagnostic clue for LCH in this patient (**b**); Acrodermatitis enteropathic from zinc deficiency; the cutaneous findings show lesions in the diaper area and other sites on the axillar and acral area (**c**)

One patient diagnosed with epidermolysis bullosa (EB; Fig. 4a) also presented with an erosive lesion in the diaper area resembling IDD. However, the fact that the rash had been present since birth and concurrent bullous lesions at sites outside of the diaper area especially pressure/ trauma associated lesions were clues that led to this diagnosis [14, 15].

Figure 4b shows a diaper rash in a patient diagnosed with LCH. Although the cutaneous lesion was superficially similar to IDD, closere inspection revealed petechial hemorrhagic lesions (arrow signs). The finding of petechiae/ hemorrhagic cutaneous signs was a clue that allowed for the early detection of LCH in this patient [16]. Moreover, there were other cutaneous lesions (such as hemorrhagic seborrheic dermatitis-like lesions) on the patient’s scalp, petechiae hemorrhage on the trunk, and hepatosplenomegaly, which supported the diagnosis of LCH. Skin biopsy of the scalp lesions was performed and the diagnosis of LCH was histopathologically confirmed by positive S100 and CD1a staining.

Figure 4c shows another presentation of diaper rash with a final diagnosis of acrodermatitis enteropathica from zinc deficiency. The patient had diarrhea before developing any cutaneous signs. Although IDD was the initial diagnosis when the diaper rash was first detected, cutaneous findings on the axillar and acral area eventually led to the final diagnosis. The patient also had alopecia, which is one of the clinical features of zinc deficiency. Although we were unable to confirm serum zinc level directly, the diagnosis was supported by the patient’s low serum alkaline phosphatase levels (41 U/L) The patient’s diarrhea and cutaneous lesions dramatically improved after 1 week of zinc supplementation.

**Fig. 5:**
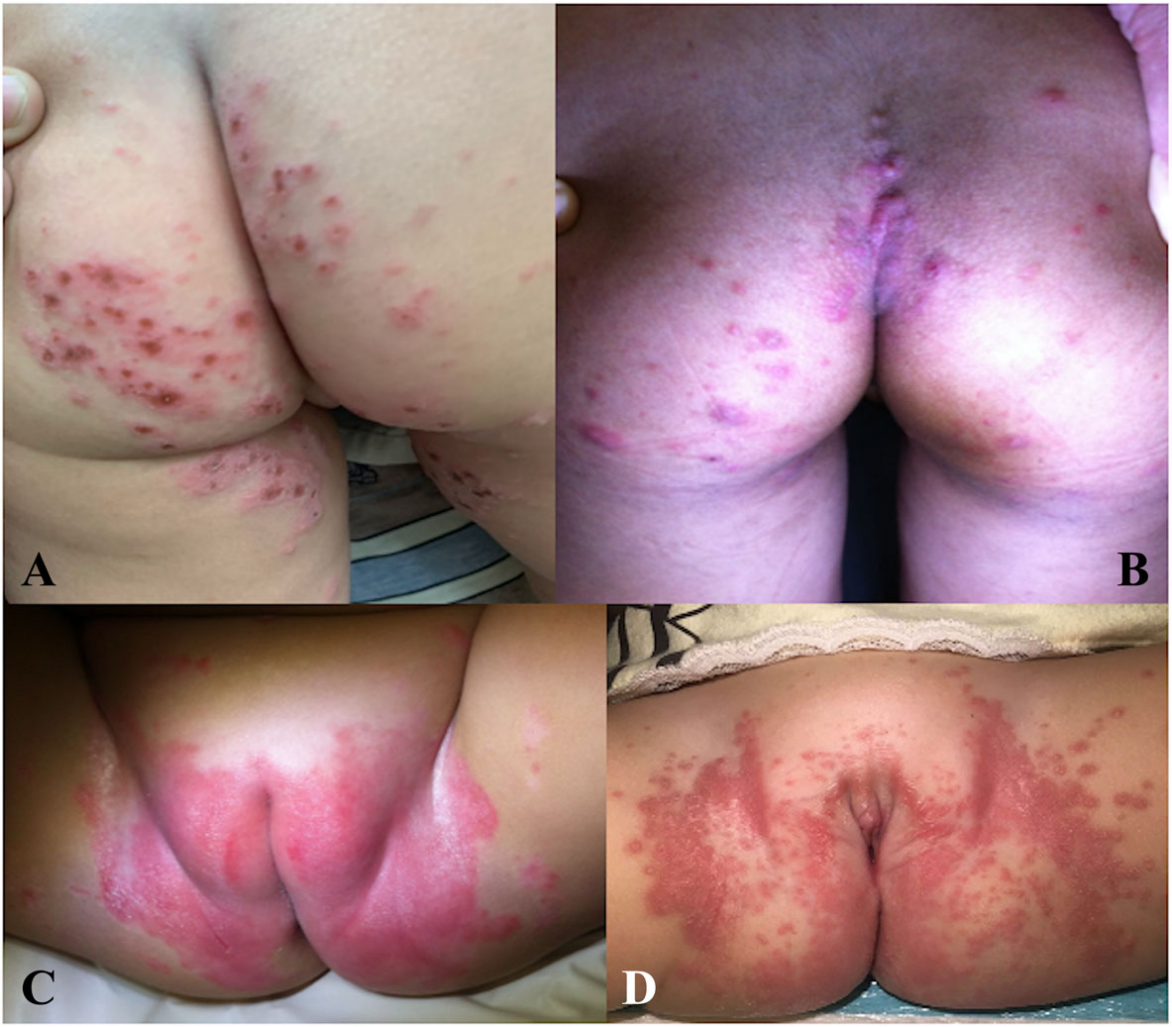
Variation of diaper rashes in the diaper area; vesicular eruptions on the buttock region in a patient with hand-foot-mouth disease (**a**), multiple itchy scabitic nodules on the buttock area (**b**), sharply demarcated erythematous plaque of paoriasis in the diaper area (**c**), classic satellite lesions of cutaneous candidiasis in the diaper area (**d**)

Hand-foot-mouth (HFM) disease was another systemic condition in the study population of which the initial presentation was diaper rash. Patients with HFM disease usually present with fever, classic cutaneous lesions of the papules or vesicular eruptions on the palms and soles, and painful vesicles on the lips and oral mucosa [17]. In the present study, there was one patient whose chief complaint was rashes in the diaper area (Fig. 5a). A diagnosis of HFM disease was made only after rashes on the palms, soles, and lips, which had not been detected upon initial examination, had progressed. This emphasizes the importance of observing rashes for any changes.

Fig. 5b shows a case of scabies infestation. The patient presented with the chief complaint of severe itching in the buttock area, especially at night. Physical examination showed multiple erythematous nodules in the diaper area (Fig. 5b). Cutaneous lesions of erythematous nodules were also observed on the patient’s axillar and peri-umbilicus concurrent with the presence of rashes in the diaper area. Definite diagnosis was made based on a fresh smear from a cutaneous lesion scraping that revealed scabies mites.

Psoriasis can also present in the diaper area, a condition called inverse psoriasis [18–20]. One patient in our study presented with sharply demarcated erythematous plaque in the diaper area (Fig. 5c) and was diagnosed with inverse psoriasis. The same patient also had concurrent cutaneous lesions on the scalp and axillar which was a clue that led to this diagnosis. Histopathology from skin biopsy confirmed the diagnosis of psoriasis in this patient.

Cutaneous candidiasis in the diaper area was the third most common diagnosis in the study population (25 cases; 8.33%). It generally manifested as widespread erythematous plaques with discrete red satellite papules (Fig. 5d). This cutaneous finding is commonly seen in the diaper region and is one of a number of conditions aggravated by wearing a diaper [7, 21, 22]. In addition to applying topical antifungal medication, one treatment modality is, thus, frequent diaper changing in order to reduce moisture and heat which can aggravate this condition.

The cases mentioned above were only some of the interesting findings from this prospective study. Some diagnoses were delayed because of a failure to notice specific diagnostic clues. One major detectable factor that was found to be significantly related to the diagnosis of cutaneous lesions other than IDD was the concurrent presence of cutaneous lesions at other sites (Table 2). Thus, complete history taking and full physical examination of the entire the body are important when such lesions are observed.

One of the strengths of the study was its prospective design, which allowed for the collection of all necessary information including photographs of the patients for research purposes. Moreover, we examined a wide variety of diaper rashes and found that there were many cases in which rashes other than IDD had manifestations in the diaper area. Detailed examination should thus be conducted prior to diagnosis as part of routine pediatric practice.

## Conclusions

The present study pointed out that many systemic conditions initially presented as rashes in the diaper area and may be misdiagnosed as IDD. Our study found out that the simultaneous presence of cutaneous lesions at other sites was a clue that indicated systemic diseases other than IDD, (*P* < 0.001). Thus, when the patient’s chief complaint is a rash in the diaper area, complete physical examination for such lesions at other sites should be conducted to avoid misdiagnosis. Observation of cutaneous changes, complete history taking, and full repeat physical examination are also imperative in routine pediatric practice to prevent unnecessary diagnostic errors.

## Data Availability

The datasets used and/or analysed during the current study are available from the corresponding author on reasonable request.
